# Epidemiological characteristics of hand, foot, and mouth disease in Shandong, China, 2009–2016

**DOI:** 10.1038/s41598-017-09196-z

**Published:** 2017-08-21

**Authors:** Jing Wang, Tao Hu, Dapeng Sun, Shujun Ding, Michael J. Carr, Weijia Xing, Shixue Li, Xianjun Wang, Weifeng Shi

**Affiliations:** 1 School of Public Health, Shandong University, Jinan, Shandong China; 2 Shandong Universities Key Laboratory of Etiology and Epidemiology of Emerging Infectious Diseases, Taishan Medical College, Taian, Shandong China; 3 Department of Viral Infectious Diseases Control and Prevention, Shandong Center for Disease Control and Prevention, Jinan, Shandong Province China; 4 Shandong Provincial Key Laboratory of Communicable Disease Control and Prevention, Jinan, Shandong Province China; 5 Global Station for Zoonosis Control, Global Institution for Collaborative Research and Education (GI-CoRE), Hokkaido University, Sapporo, Japan; 6 National Virus Reference Laboratory, University College Dublin, Dublin 4, Ireland; 7 School of Public Health, Taishan Medical College, Taian, Shandong China

## Abstract

In the past decade, hand, foot, and mouth disease (HFMD) has posed a serious threat to childhood health in China; however, no epidemiological data from large HFMD epidemics have been described since 2013. In the present study, we described the epidemiological patterns of HFMD in Shandong province during 2009–2016 from a large number of symptomatic cases (n = 839,483), including >370,000 HFMD cases since 2013. Our results revealed that HFMD activity has remained at a high level and continued to cause annual epidemics in Shandong province from 2013 onwards. Although the incidence rate was significantly higher in urban areas than in rural areas, no significantly higher case-severity and case-fatality rates were found in urban areas. Furthermore, the seventeen cities of Shandong province could be classified into three distinct epidemiological groups according to the different peak times from southwest (inland) to northeast (coastal) regions. Notably, a replacement of the predominant HFMD circulating agent was seen and non-EVA71/Coxsackievirus A16 enteroviruses became dominant in 2013 and 2015, causing approximately 30% of the severe cases. Our study sheds light on the latest epidemiological characteristics of HFMD in Shandong province and should prove helpful for the prevention and control of the disease in Shandong and elsewhere.

## Introduction

The first case of hand, foot, and mouth disease (HFMD) was first described clinically in New Zealand and Canada in 1957^1^. HFMD is a viral infectious disease associated with different types of positive polarity, single-stranded RNA virus members of the family *Picornaviridae*, genus *Enterovirus* (EV), notably enterovirus A71 (EVA71) and Coxsackievirus A16 (CVA16)^1–4^. Children under five are disproportionately affected populations with a febrile presentation and vesicular exanthema on the hands, feet, mouth and buttocks. HFMD syndrome is generally mild and self-limiting; however, severe neurologic sequelae can occur^5^. Person-to-person transmission is by direct contact with feces, saliva or respiratory secretions or via indirect contact with contaminated objects serving as fomites.

Over the last decade, many severe HFMD outbreaks have been reported in East and Southeast Asia, which have become a serious public health issue in the affected countries^6–9^. In China, the first large-scale outbreak of HFMD was reported in Linyi, Shandong Province in 2007, which resulted in 1149 diagnosed cases and 3 deaths^10^. In 2008, a HFMD outbreak, in Fuyang, Anhui Province, led to 353 severe cases and 22 deaths^11^. To better respond to the HFMD outbreaks, in 2008, HFMD was classified as a statutorily notifiable infectious disease by the Ministry of Health of China^12^. Since 2008, more than one million HFMD cases were reported to National Surveillance Systems every year, including thousands of infant deaths^13^. Further analysis revealed that the seasonal pattern of HFMD was different in northern (latitude range 35.5°–46.2°N, including Tibet) and southern (19.5°–34.8°N) China^13^. In northern China, only one annual outbreak peak was observed in June from 2008 to 2012. In contrast, in southern China, two semi-annual outbreak peaks occurred, with one in spring-summer and the other in autumn.

The potential association between epidemiological and meteorological variables has been previously described in different regions in the Asia-Pacific region: such as Guangdong (2010–2012)^14^, Hong Kong (2000–2009)^15^, Taiwan (2012–2014)^16^, Shandong (2008–2012)^17^, Shanxi (2009–2013)^18^ and Beijing (2008–2011)^19^ in China, as well as in Japan (2000–2010)^20^ and Singapore (2001–2008)^21^. Despite application of different models, these prior studies reached a consensus that there is a strong association between HFMD incidence and meteorological variables; however, in certain different geographic regions, the meteorological variables contributed less significantly to the incidence of HFMD.

Shandong is a coastal province at the juncture of north and south China and is one of the most HFMD-stricken provinces. Since the first large-scale HFMD outbreak in Linyi in 2007^10^, approximately 100,000 HFMD cases were reported annually, and the accumulative HFMD cases in Shandong from 2007 to 2011 ranked among the top five of the 31 Chinese provinces^22^. The predominant circulating enterovirus serotype during 2007–2011 was EVA71, with CVA16 predominating in Shandong province in 2010^22^; however, no large-scale epidemiological studies of HFMD in mainland China have been reported since 2013. In the present study, we characterize the epidemiology of HFMD in Shandong province, focusing on age, gender, virological, seasonal and geographical patterns of HFMD during the period from 2009 to 2016.

## Results

**Figure 1:**
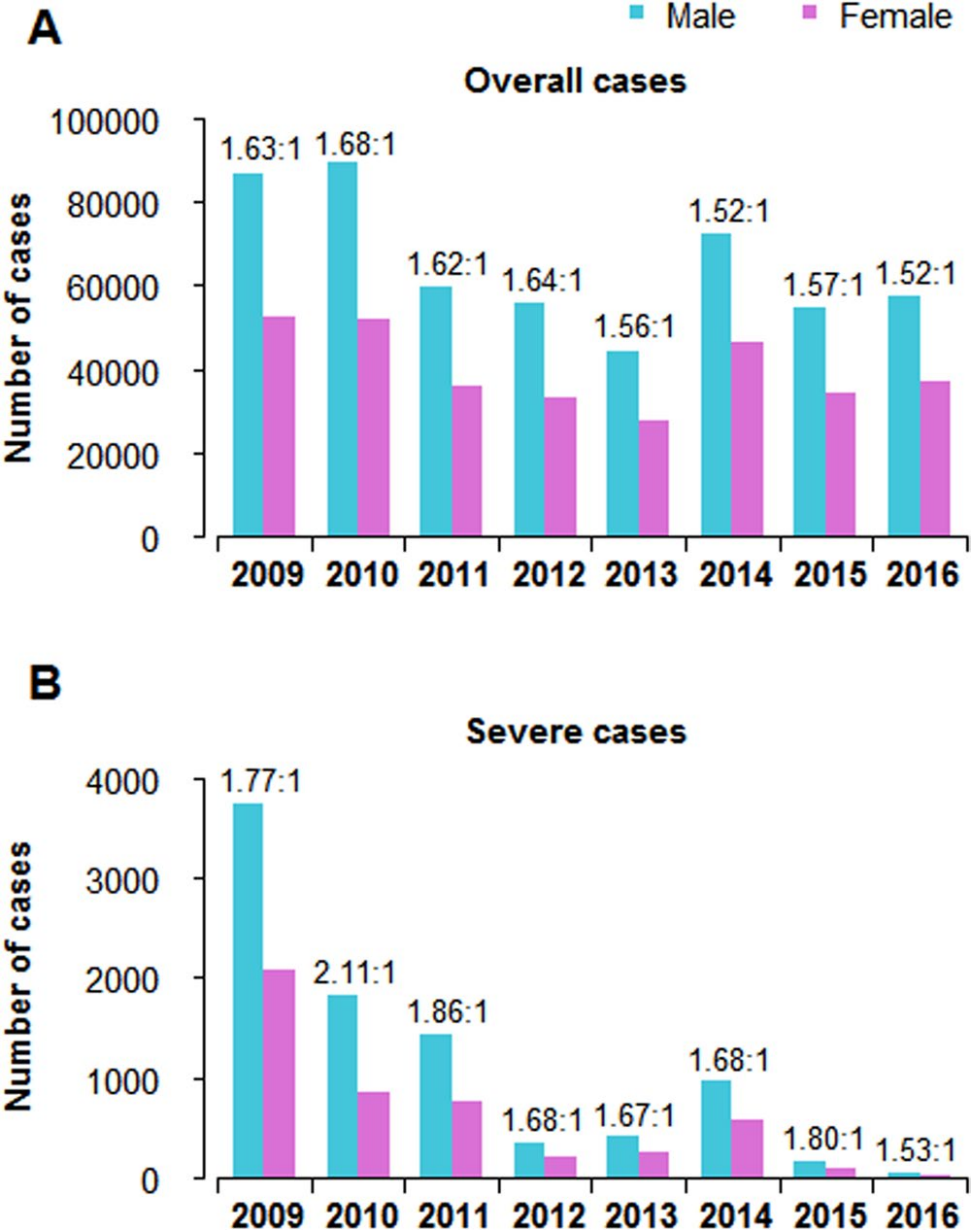
Sex distribution and clinical severity of overall cases of HFMD in Shandong province, 2009–2016. (**A**) Sex distribution of probable and laboratory-confirmed cases. (**B**) Sex distribution of severe cases.

In Shandong province, symptomatic HFMD cases (n = 839,483) were reported to the surveillance system from 2009 to 2016, of which 48,133 (5.73%) cases were confirmed by laboratory testing. Of these, 13,732 (1.64%) cases developed severe complications, of which 75 cases had a fatal outcome (case-fatality rate: 0.01%), with a severe case-fatality rate of 0.55%. The majority of HFMD cases were aged under five years (90.15%), with the median age of 2.4 years (interquartile range (IQR): 1.5–3.8 years). The number of reported cases was higher in males than females, and the sex ratio between male and female cases was approximately 1.6:1. The sex ratio was similar among mild and severe cases; male cases always had a higher proportion of cases than female cases, even if the total number of cases changed across different years (Fig. 1).

The incidence rates of HFMD cases were higher in 2009 and 2010, reaching a maximum in 2010 with nearly 1500 cases per million person-years (Table 1). The incidence rates then declined to 754 cases per million person-years in 2013; however, the incidence rate increased again in 2014 and was >900 cases per million person-years during 2015–2016. The incidence rates of severe cases decreased dramatically from 60.61 cases per million persons in 2009 to 0.08 cases per million persons in 2016, yet an increase in the incidence rate of severe cases was observed in 2014 (Table 2). Similarly, the mortality rate of HFMD cases in 2009 was 0.47 cases per million persons, whereas no fatal cases were reported in 2016 (Table 3).

**Table 1:** Incidence rates (per million) of HFMD by age groups in Shandong province, 2009–2016.

Age groups	2009	2010	2011	2012	2013	2014	2015	2016
<6 months	2825.5 (2669.9–2981.2)	2089.0 (1955.2–2222.9)	1727.5 (1605.8–1849.2)	1928.3 (1799.7–2056.9)	2017.6 (1886.1–2149.1)	2388.1 (2245.0–2531.2)	2738.5 (2585.3–2891.7)	1944.0 (1814.9–2073.1)
6–11 months	46520.1 (45940.7–47099.5)	38114.3 (37589.9–38638.7)	23579.4 (23166.9–23991.8)	19602.8 (19226.7–19978.9)	22086.0 (21686.8–22485.3)	22865.6 (22459.4–23271.8)	29086.8 (28628.6–29544.9)	15042.1 (14712.6–15371.6)
12–23 months	40790.2 (40408.7–41171.6)	36839.4 (36476.9–37201.9)	24129.9 (23836.5–24423.3)	22594.7 (22310.8–22878.7)	20843.2 (20570.5–21115.9)	30652.3 (30321.6–30983.0)	24698.3 (24401.4–24995.1)	26809.3 (26500.0–27118.5)
24–59 months	17956.7 (17811.6–18101.8)	21026.7 (20869.7–21183.6)	14206.6 (14077.6–14335.7)	13187.3 (13062.9–13311.6)	9280.4 (9176.1–9384.7)	17897.2 (17752.4–18042.0)	11150.4 (11036.1–11264.7)	13864.6 (13737.1–13992.1)
5–9 years	1801.6 (1764.2–1838.9)	2082.2 (2042–2122.3)	1793.3 (1756.1–1830.5)	1768.7 (1731.8–1805.7)	1133.7 (1104.1–1163.3)	2399.2 (2356.1–2442.3)	1472.0 (1438.3–1505.8)	2026.6 (1987.0–2066.2)
10–14 years	234.1 (220.4–247.8)	181.1 (169–193.1)	145.7 (134.8–156.5)	161.4 (150–172.8)	126.6 (116.5–136.7)	208.1 (195.2–221.1)	159.9 (148.6–171.3)	202.5 (189.7–215.2)
≥15 years	4.7 (4.2–5.1)	5.4 (4.9–5.9)	4.4 (3.9–4.8)	5.3 (4.8–5.8)	6.0 (5.5–6.5)	8.6 (8.0–9.3)	8.4 (7.8–9.1)	6.6 (6.0–7.1)
Total	1453.7 (1446–1461.3)	1476.7 (1469–1484.4)	1000.4 (994.1–1006.7)	927.4 (921.3–933.5)	754.1 (748.6–759.6)	1237.2 (1230.1–1244.2)	925.0 (918.9–931.1)	989.1 (982.8–995.4)

**Table 2:** Incidence rates (per million) of severe cases of HFMD by age groups in Shandong province, 2009–2016.

Age groups	2009	2010	2011	2012	2013	2014	2015	2016
<6 months	281.2 (232.1–330.3)	78.1 (52.2–104.0)	80.3 (54.1–106.6)	42.4 (23.3–61.5)	46.9 (26.8–66.9)	133.9 (100.0–167.8)	13.4 (2.7–24.1)	2.2 (0.0–6.6)
6–11 months	2783.8 (2642.0–2925.5)	764.5 (690.2–838.8)	693.1 (622.4–763.8)	124.0 (94.1–153.9)	206.6 (168.0–245.2)	257.3 (214.2–300.4)	52.6 (33.1–72.1)	13.1 (3.4–22.9)
12–23 months	2141.6 (2054.2–2229.0)	980.7 (921.6–1039.9)	725.3 (674.5–776.2)	153.2 (129.9–176.6)	235.9 (206.9–264.9)	492.2 (450.3–534.1)	77.1 (60.5–93.7)	39.9 (28.0–51.9)
24–59 months	524.5 (499.7–549.3)	330.1 (310.5–349.8)	270.3 (252.5–288.1)	78.4 (68.8–88.0)	73.5 (64.2–82.8)	215.7 (199.8–231.6)	31.1 (25.1–37.2)	7.6 (4.6–10.6)
5–9 years	32.6 (27.6–37.6)	16.7 (13.1–20.3)	22.1 (18.0–26.3)	6.0 (3.9–8.2)	7.6 (5.2–10.1)	19.1 (15.3–23.0)	2.8 (1.3–4.3)	0.2 (0.0–0.6)
10–14 years	2.1 (0.8–3.4)	2.1 (0.8–3.4)	2.5 (1.1–3.9)	0.4 (−0.2–1.0)	0.6 (-0.1–1.3)	1.7 (0.5–2.8)	0.0 (0.0–0.0)	0.0 (0.0–0.0)
≥15 years	0.0 (0.0–0.0)	0.0 (0.0–0.1)	0.0 (0.0–0.0)	0.0 (0.0–0.0)	0.0 (0.0–0.0)	0.0 (0.0–0.0)	0.0 (0.0–0.0)	0.0 (0.0–0.0)
Total	60.6 (59.1–62.2)	27.9 (26.9–29.0)	22.9 (22.0–23.9)	5.6 (5.2–6.1)	7.0 (6.4–7.5)	16.1 (15.3–16.9)	2.4 (2.1–2.7)	0.8 (0.6–1.0)

**Table 3:** Incidence rates of fatal cases of HFMD by age groups in Shandong province, 2009–2016.

Age groups	2009	2010	2011	2012	2013	2014	2015	2016
<6 months	6.7 (0.0–14.3)	0.0 (0.0–0.0)	0.0 (0.0–0.0)	0.0 (0.0–0.0)	0.0 (0.0–0.0)	0.0 (0.0–0.0)	2.2 (0.0–6.6)	0.0 (0.0–0.0)
6–11 months	43.2 (25.5–60.9)	5.6 (0.0–12.0)	3.8 (0.0–9.0)	0.0 (0.0–0.0)	3.8 (0.0–9.0)	1.9 (0.0–5.6)	1.9 (0.0–5.6)	0.0 (0.0–0.0)
12–23 months	15.8 (8.3–23.3)	1.9 (0.0–4.4)	1.9 (0.0–4.4)	0.0 (0.0–0.0)	3.7 (0.1–7.4)	0.9 (0.0–2.7)	0.0 (0.0–0.0)	0.0 (0.0–0.0)
24–59 months	0.6 (0.0–1.5)	0.9 (0.0–2.0)	1.5 (0.2–2.9)	0.6 (0.0–1.5)	0.0 (0.0–0.0)	0.0 (0.0–0.0)	0.3 (0.0–0.9)	0.0 (0.0–0.0)
5–9 years	0.0 (0.0–0.0)	0.0 (0.0–0.0)	0.0 (0.0–0.0)	0.0 (0.0–0.0)	0.0 (0.0–0.0)	0.0 (0.0–0.0)	0.0 (0.0–0.0)	0.0 (0.0–0.0)
10–14 years	0.0 (0.0–0.0)	0.0 (0.0–0.0)	0.0 (0.0–0.0)	0.0 (0.0–0.0)	0.0 (0.0–0.0)	0.0 (0.0–0.0)	0.0 (0.0–0.0)	0.0 (0.0–0.0)
≥15 years	0.0 (0.0–0.0)	0.0 (0.0–0.0)	0.0 (0.0–0.0)	0.0 (0.0–0.0)	0.0 (0.0–0.0)	0.0 (0.0–0.0)	0.0 (0.0–0.0)	0.0 (0.0–0.0)
Total	0.5 (0.3–0.6)	0.1 (0.0–0.1)	0.1 (0.0–0.2)	0.0 (0.0–0.0)	0.1 (0.0–0.1)	0.0 (0.0–0.0)	0.0 (0.0–0.1)	0.0 (0.0–0.0)

Incidence rates of HFMD had broad age-specific variation (Table 1). The incidence rates in infants aged 6–11 months and in children aged 12–23 months were comparable, ranking first and second, respectively. Furthermore, patients from these two age groups accounted for 49%, 42%, 40%, 39%, 47%, 38%, 47% and 39% of all HFMD cases from 2009 to 2016, respectively. In addition, the incidence rates of the pediatric group aged 24–59 months were also high, accounting for an average of 46.13% of all HFMD cases due to the large number of children in this age group (Table 1). Irrespective of the incidence rates of the overall cases, the incidence rates of severe cases or the mortality rates, all were lower in infants aged less than 6 months, children aged 5–14 years and adolescents aged above 14 years (Tables 2 and 3).

**Figure 2:**
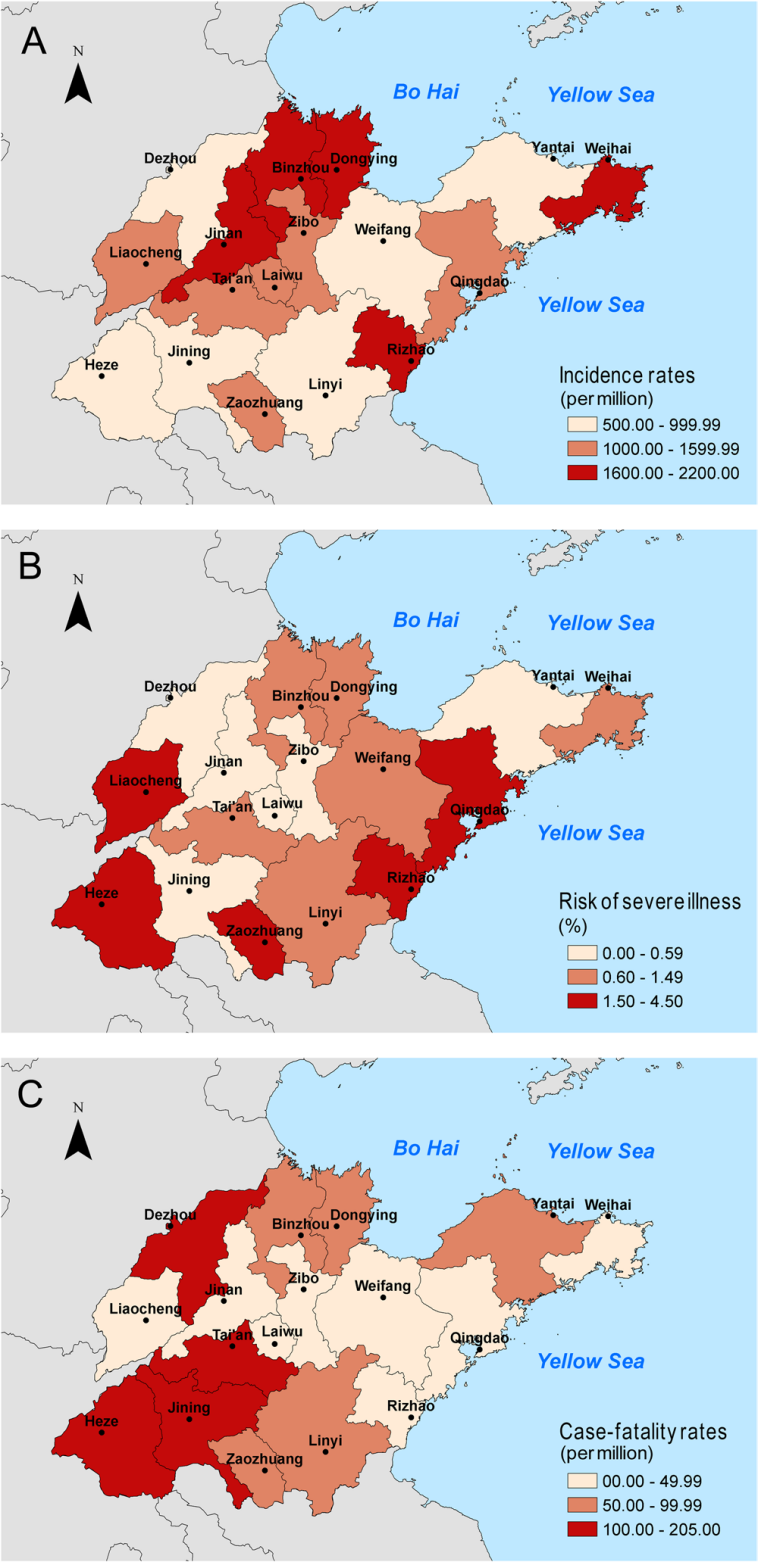
Estimated average incidence rates, case-severity risk and case-fatality ratesof all HFMD cases in 17 cities of Shandong province, 2009–2016. (**A**) Geographic distribution of average incidence rates of probable and laboratory-confirmed cases. (**B**) Geographic distribution of average case-severity risk of probable and laboratory-confirmed cases. (**C**) Geographic distribution of average case-fatality rates of probable and laboratory-confirmed cases.

There were significant differences in the incidence rates of HFMD among the cities of Shandong province (range: 500–2200 cases per million person-years, Fig. 2A). The highest average incidence rate was found in Dongying on the northern coast of Shandong province, and the lowest rate was in Jining, located inland in southwestern Shandong. The average case-severity risk varied from 0.04% to 4.32% across different cities, and the risks were generally higher in the western and southeastern regions of Shandong province (Fig. 2B). The case-fatality rates were higher in the southern and southwestern regions (Fig. 2C). The incidence rates differed according to the area of residence, and the incidence rates were always higher in urban children (*p* = 0.000, Table 4). Nevertheless, no significant difference in the case-severity rate (*p* = 0.471) and the case-fatality rate (*p* = 0.069) between urban and rural cases was found during the study period (Table 4).

**Table 4:** Clinical severity and residence type of HFMD cases in Shandong province, 2009–2016.

Clinical severity	Residence type	2009	2010	2011	2012	2013	2014	2015	2016
Incidence rates (per million)	Urban	2044.0 (2027.3–2060.6)	2643.9 (2625.0–2662.9)	1764.3 (1748.9–1779.8)	1749.4 (1734.0–1764.8)	1347.1 (1333.6–1360.6)	2318.6 (2300.9–2336.3)	1929.4 (1913.3–1945.6)	2083.8 (2067.0–2100.6)
	Rural	1205.3 (1197.1–1213.6)	985.6 (978.1–993.1)	679.0 (672.8–685.2)	581.6 (575.8–587.3)	504.7 (499.3–510.1)	782.3 (775.6–789.0)	502.5 (497.1–507.8)	528.6 (523.1–534.1)
Case severity risks (%)	Urban	3.25% (3.10–3.39%)	1.45% (1.37–1.54%)	2.11% (1.99–2.24%)	0.64% (0.57–0.71%)	1.07% (0.97–1.17%)	1.72% (1.62–1.82%)	0.24% (0.20–0.28%)	0.07% (0.05–0.09%)
	Rural	4.83% (4.68–4.98%)	2.39% (2.27–2.50%)	2.48% (2.34–2.63%)	0.57% (0.49–0.64%)	0.76% (0.67–0.85%)	0.78% (0.70–0.85%)	0.30% (0.24–0.36%)	0.10% (0.07–0.14%)
Case fatality rates (%)	Urban	0.009% (0.001–0.016%)	0.004% (0.000–0.009%)	0.002% (0.000–0.006%)	0.002% (0.000–0.006%)	0.003% (0.000–0.008%)	0.002% (0.000–0.005%)	0.000% (0.000–0.000%)	0.000% (0.000–0.000%)
	Rural	0.049% (0.034–0.064%)	0.008% (0.001–0.014%)	0.017% (0.005–0.030%)	0.003% (0.000–0.008%)	0.015% (0.002–0.028%)	0.002% (0.000–0.006%)	0.009% (0.000–0.019%)	0.000% (0.000–0.000%)

**Figure 3:**
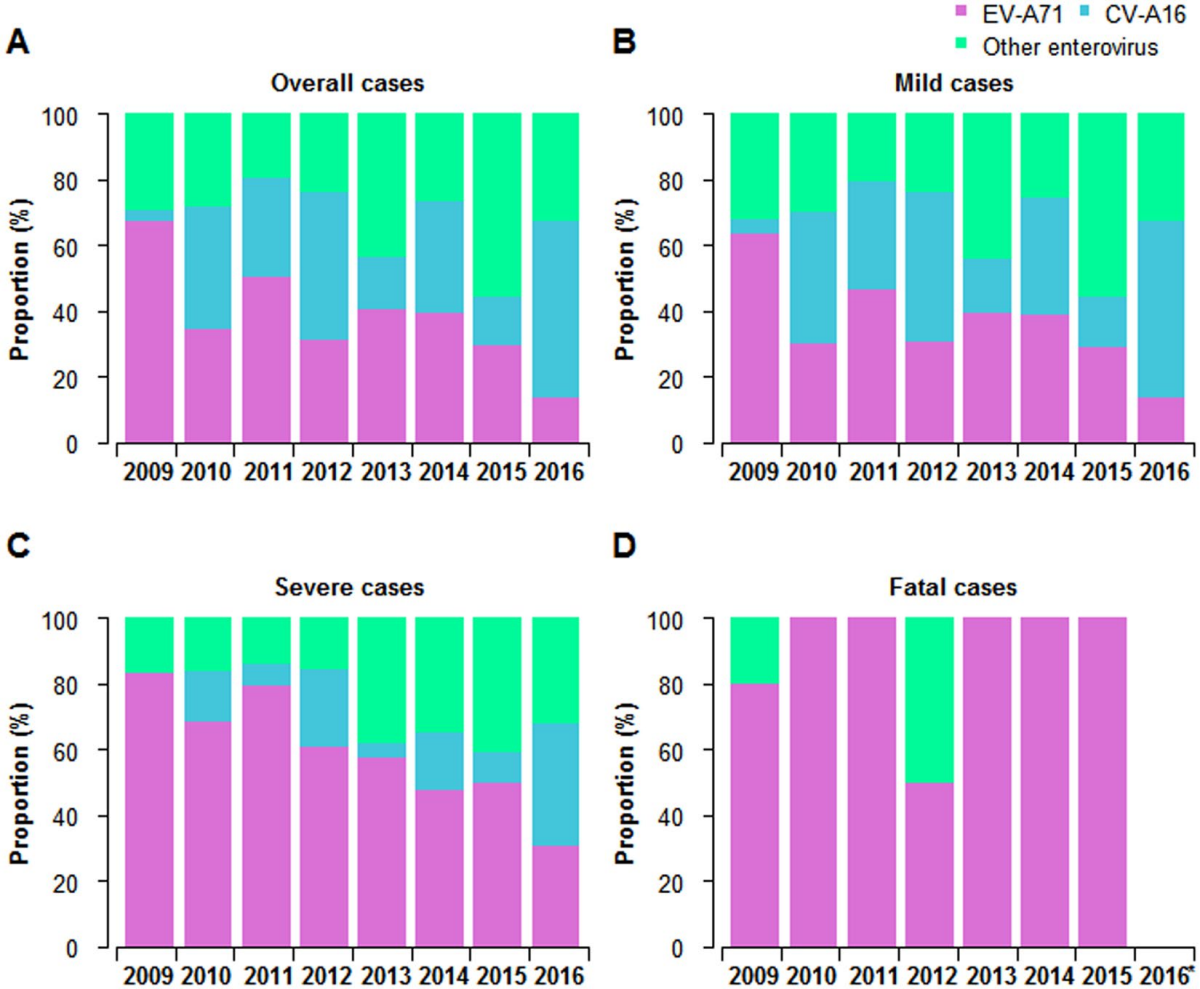
Proportion of enterovirus serotypes in laboratory-confirmed cases of HFMD by clinical severity in Shandong province, 2009–2016. (**A**) Based on all the probable and laboratory-confirmed cases. (**B**) Based on mild cases. (**C**) Based on severe cases. (**D**) Based on fatal cases.

In laboratory-confirmed cases, the predominant circulating enterovirus serotypes associated with HFMD changed each year (Fig. 3A). EVA71 predominated in 2009, 2011 and 2014, accounting for 67%, 50% and 39% of all laboratory-confirmed HFMD cases, respectively. CVA16 predominated in 2010, 2012 and 2016, accounting for 37%, 45% and 53% of all laboratory-confirmed HFMD cases, respectively. Other non-EVA71/CVA16 enteroviruses continued to circulate at a low level prior to 2013 and represented the predominant pathogens in 2013 (44%) and 2015 (56%), respectively. The proportions of different serotypes in mild cases were similar (Fig. 3B). However, EVA71 was most frequently detected in severe and fatal cases, accounting for 60% of severe cases (range across year: 30–83%) and 90% of fatal cases (range across year: 50–100%, Fig. 3C and D). However, in 2016, CVA16 predominated for the first time in severe cases (38%, Fig. 3C). It should also be noted that since 2013, severe cases attributable to other enteroviruses accounted for more than 30% of all severe cases (Fig. 3C).

**Figure 4:**
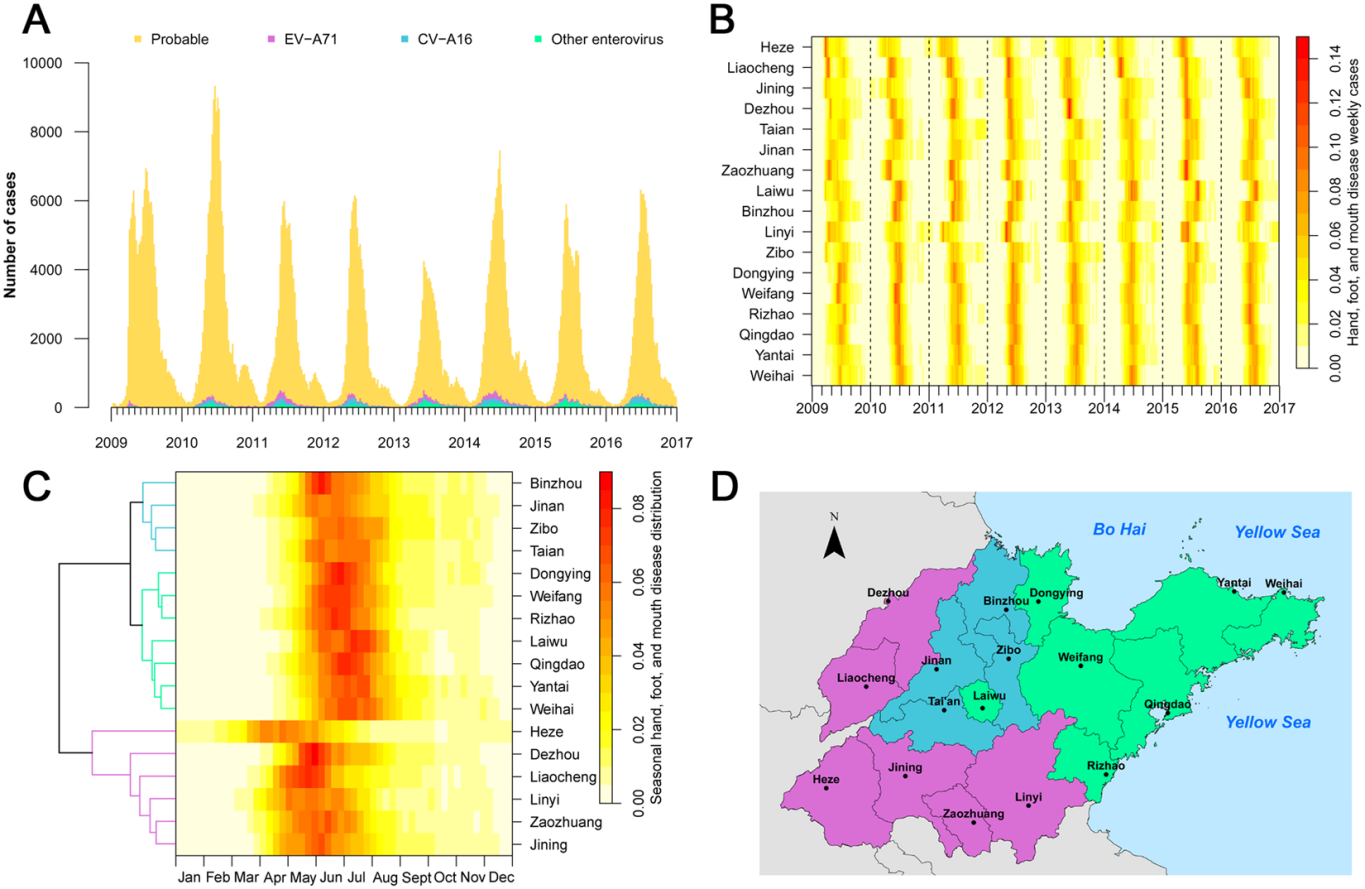
Heatmap of surveillance data for HFMD by city and HFMD epidemiological region, 2009–2016. (**A**) HFMD epidemic curve based on the number of weekly reported cases. (**B**) Time series of weekly reported cases of HFMD standardized by the number of annual cases. The cities were ordered by longitude from the westernmost (top) to the easternmost (bottom). (**C**) Clustering analysis of seasonal distribution of HFMD cases, plotted as the median value of proportion of cases in each week of the year from 2009 to 2016. (**D**) Classification of the epidemiological regions by clustering analysis.

The incidence of HFMD in Shandong province showed a major peak each year, and the incidence began to increase in March and reached a peak during May to July period (Fig. 4A). However, a smaller autumnal peak (around week 45) was evident in 2010, 2011, 2014 and 2015. At the city level across Shandong, the incidence did not reach a peak simultaneously. The incidence began to increase and peaked early in the southwestern region, and then in the northeastern region (Fig. 4B and C). The seventeen cities of Shandong province could be clustered into three groups according to their trend of increasing HFMD incidence. The first included Heze, Dezhou, Liaocheng, Linyi, Zaozhuang and Jining; the second comprised Binzhou, Jinan, Zibo and Taian; and, finally, the third consisted of Dongying, Weifang, Rizhao, Laiwu, Qingdao, Yantai and Weihai (Fig. 4C and D). The average peak time of the three groups of cities during 2009–2016 was the 20^th^, 25^th^, and 27^th^ week (*p* < 0.0001), respectively.

## Discussion

Since 2008, HFMD has posed a serious threat to childhood health and has become one of the leading causes of childhood mortality in China^13^. Although there have been a number of epidemiological studies in China^13–19^, no epidemiological data of a large number of HFMD patients have been described since 2013. Shandong province is one of the five most stricken provinces of HFMD^13, 22^. In the present study, we describe the HFMD burden and epidemiological patterns of HFMD in Shandong province between 2009–2016, including more than 0.37 million HFMD cases reported to the national enhanced surveillance system since 2013, which is the most comprehensive dataset describing the latest epidemiology of HFMD in China.

The HFMD incidence rates of Shandong province ranged from 754 in 2013 to 1454 in 2010 per million person-years, with an annual average incidence rate of 1095 per million person-years. The highest incidence rate in Shandong province was observed in 2012, which is generally in accordance with the situation of mainland China^13^. The annual incidence rates in 2011 and 2012 of Shandong province (>1400 cases per million person-years) were much lower than those of Guangzhou (>4000 cases per million person-years in 2011 and 2012)^23^. Since 2013, the annual incidence rates of Shandong province decreased and were comparable with that reported for Beijing (~940 cases person-years during 2008–2011)^19^. However, although the HFMD incidence rates decreased after 2012, the viral agents responsible continued circulating at a high level in Shandong, infecting an average of approximately 0.1 million children annually with the majority aged between 6–59 months. With regards to the gender ratio of the incidence rates, the results obtained from Shandong province and the rest of China were comparable, with 1.6 times higher incidence in boys than in girls^13^. Therefore, intervention measures, such as vaccine administration for both genders should be considered prior to attending crèche facilities and also targeted improvements in hygiene, particularly for boys aged 6–59 months, which would likely serve to reduce HFMD cases.

Although the incidence rates in the urban areas were significantly higher than those in the rural areas, both the case-severity and case-fatality rates in rural areas were similar with those of the urban areas. Remarkably, most of the severe cases were found in central areas of Shandong; however, the case-fatality rates in western Shandong were the highest, where the health and medical conditions are less developed than the remaining regions of Shandong province. These results suggest that improved healthcare facilities with timely diagnosis and treatment may be helpful to reduce severe and fatal cases associated with HFMD infections.

In addition, to explore the possible role of demographic factors, we collected information about the birth rate and number of newborns, health expenditure, numbers of hospitals and medical doctors in Shandong province in the period 2009 and 2014. Except for 2014 (14.24‰), the birth rate of Shandong province was stable between 2009 and 2015, with an average of 11.79‰. However, the health expenditure per person, the proportion of health expenditure over GDP, the numbers of hospitals, health facilities and medical doctors have greatly increased in Shandong province during the study period. Therefore, it seems that the change of incidence rates of HFMD is not positively correlated to the birth rate and is less affected by improvements in medical care. Notably, as China has introduced a “two child per family” policy, it is likely that the country will face an ongoing HFMD burden in the years ahead.

Large-scale outbreaks of Coxsackievirus A6 (CVA6)^24–26^ and Coxsackievirus A10 (CVA10)^27, 28^ have been documented in Asian-Pacific regions and also in Europe in the past few years. Although EVA71 was the predominant serotype among the circulating HFMD agents in China from 2008 to 2012^13^, the predominant enterovirus in Shandong province shifted between EVA71, CVA16 and other enteroviruses almost annually. In particular, other enteroviruses became predominant in 2013 and 2015, which was not previously seen before in Shandong^22^. As neutralizing antibodies induced by EVA71, CVA16, CVA6 and CVA10 would only provide protective immunity for their respective serotypes and are not expected to elicit heterotypic immunity to reduce the incidence of infections caused by other serotypes of enteroviruses^29–31^, this highlights the need for further detailed molecular epidemiological analysis of non-EVA71/CVA16 enteroviruses in circulation to determine optimal vaccine strategies in Shandong province and the requirements for a multivalent HFMD vaccine.

It should be noted that a Vero cell-based EVA71 inactivated vaccine adjuvanted with aluminum hydroxide has been available in China, including Shandong province, since licensing was approved in December 2015. However, to date, here have been no reports regarding how many children have received this vaccine and, to the best of our knowledge, very few children have, as yet, received prophylaxis. Therefore, we consider that the commercialized EVA71 vaccine plays a very limited role at present in limiting the incidence of EVA71.

Only one major annual epidemic peak was observed in Shandong province, which was consistent with the epidemiological characteristics of HFMD in northern China^13^. It has been reported that the national annual HFMD incidence rates peaked earlier in southern than in northern China^13, 32^. The HFMD incidence rates in Shandong province peaked from southwest (inland) to northeast (coastal) regions and the seventeen cities of Shandong province could be classified into three groups based on the different peak times of HFMD disease incidence. According to recent reports regarding the phase III trial of the EVA71 vaccine, the EVA71 neutralizing antibody titer was greater than 1:8 for > 98% of the participants after day 56 and may decline by half after the first six months^33, 34^. Therefore, we suggest that the optimal timing for vaccine administration for children in these regions could be mid-January, because the peak season of HFMD in western and southwest Shandong usually starts from early April. For middle and eastern Shandong, the peak season usually starts from late April and the optimal timing for vaccine administration should be no later than mid-February. However, for Heze, the peak season was found to start from early March and the optimal timing for vaccine administration should be late December of the previous year.

The association between the incidence rates of HFMD and meteorological factors has been extensively studied^14–21^. Average temperature^14, 15, 18–21^, rainfall^21^, average wind speed and relative humidity (RH) over the prior two weeks^20, 35^ and precipitation^36^ have been associated with the incidence rates of HFMD. Although temperature was found to be the most influential meteorological factor in different regions, its effect may differ. For example, every 1 °C increase in maximum temperature above 32 °C elevated the risk of HFMD incidence by 36% in Singapore^21^. In addition, meteorological factors have also been reported to be associated with the HFMD incidence in Shandong^35^. However, based on current evidence, meteorological factors play different roles in different regions, especially in regions with large weather difference, such as, Singapore^21^ and Beijing^19^. Therefore, we think that meteorological factors are clearly not the sole determinants of HFMD incidence rates. It is likely that yearly fluctuations in the rates of herd immunity against different enteroviruses in children would be a major determinant and that the decrease of the numbers of susceptible children - by targeted intervention with safe, well-tolerated and efficacious multivalent vaccines - would be predicted to substantially decrease HFMD-associated morbidity and mortality.

In summary, we describe the latest epidemiological characteristics of HFMD in one the most seriously affected Chinese provinces. Our findings show that HFMD continued to cause annual epidemics since 2013 and, in addition, no significantly higher case-severity and case-fatality rates were found in rural areas compared to urban centers. Slightly different HFMD peak times were observed among the cities of Shandong, suggesting an optimal timing for vaccine administration needs careful consideration. A dynamic change of the predominant HFMD-associated agent was observed in Shandong province and non-EVA71/CVA16 enteroviruses became predominant in recent years, which will potentially complicate efforts to administer multivalent HFMD vaccines. Our study sheds light on the latest epidemiological characteristics of HFMD in Shandong province and should prove helpful for the prevention and control of HFMD-associated disease in Shandong and elsewhere.

## Materials and Methods

### Ethics statement

The ethical approval was given by Ethics Review Committee of the Shandong Center for Disease Control and Prevention, and the study was conducted in compliance with the principles of the Declaration of Helsinki. Written informed consents for the use of their clinical samples were obtained from the sick children’s legal guardians.

### Case definitions

According to the case definitions from the guidelines for HFMD public health response, a probable case was defined as a patient with papular or vesicular rash on the hands, feet, mouth or buttocks, with or without fever^13^. A confirmed case was defined as a probable case with laboratory evidence of enterovirus infection (i.e. EVA71, CVA16, or other non-EVA71 and non-CVA16 enteroviruses) detected by RT-PCR, real-time PCR or by virus isolation. All HFMD patients presenting with only the manifestations mentioned above, either probable or confirmed, were categorized as mild cases. Patients were classified as severe cases if they experienced any neurological manifestations (e.g. aseptic meningitis, encephalitis) and/or cardiopulmonary complications (e.g. pulmonary oedema, cardiorespiratory failure) during the clinical course.

### Specimen collection

The HFMD surveillance system was established with the national HFMD surveillance system in May 2008. The specimens were collected from the first five mild, probable HFMD patients who visited the hospitals every month in each of the 137 counties or districts and from all the severe and fatal cases in Shandong province. Depending on the symptoms and clinical status of each patient, the following clinical specimens were collected: throat swab, rectal swab, feces, vesicular fluid or cerebrospinal fluid.

### Specimen testing

Specimen processing was performed by provincial or prefectural public health laboratories, as previously described^13^. Viral RNA was extracted using commercial kits^13^. The RNA extracts from each specimen were tested using a pan-enteroviral assay with specific oligonucleotide primers and probes that targeted EVA71 and CVA16 in separate assays. The test results were classified into four categories: enterovirus negative, EVA71 positive, CVA16 positive, or other enterovirus positive without further serotype identification.

### Data analysis

All the probable and laboratory-confirmed HFMD cases from Shandong province reported to the National Surveillance System between January 1, 2009 and December 31, 2016 were included in the analysis. The “incidence” is measured at a timescale of week and is equal to the probable and laboratory-confirmed cases in each age group per week. The “trend of incidence” is quantified by the change of weekly incidence during 2009–2016 in each age group. The age-specific rates of incidence, illness severity and mortality of probable and laboratory-confirmed cases were estimated. The age-specific case fatality rates, fatality rates of severe cases and the case-severity risk were also estimated. The case fatality rate was calculated by dividing the number of deaths by the number of probable and laboratory-confirmed cases. The fatality rate of severe cases was calculated by dividing the number of deaths by the number of severe cases. The case-severity risk was estimated by dividing the number of severe cases by the number of probable and laboratory-confirmed cases. *T*-testing was performed to compare the difference between groups.

To explore the temporo-spatial characteristics of HFMD in Shandong province, we classified Shandong into 17 cities based on administrative areas. The “peak time” is measured by weekly incidence and it is defined as the week in which the number of new cases reaches the incidence peak (i.e. the highest number of new cases) in a year. The epidemiological regions of HFMD were identified by hierarchical clustering of the weekly incidence rates of the 17 cities. Pheatmap embedded in the R package (http://www.r-project.org/) was applied, with the clustering distance of Euclidean and the clustering method of complete linkage.
